# Neurovascular Dysfunction in Diverse Communities With Health Disparities—Contributions to Dementia and Alzheimer’s Disease

**DOI:** 10.3389/fnins.2022.915405

**Published:** 2022-06-29

**Authors:** Napatsorn Saiyasit, Evan-Angelo R. Butlig, Samantha D. Chaney, Miranda K. Traylor, Nanako A. Hawley, Ryleigh B. Randall, Hanna V. Bobinger, Carl A. Frizell, Franklin Trimm, Errol D. Crook, Mike Lin, Benjamin D. Hill, Joshua L. Keller, Amy R. Nelson

**Affiliations:** ^1^Department of Physiology and Cell Biology, College of Medicine, University of South Alabama, Mobile, AL, United States; ^2^Department of Neurology, Eli and Edythe Broad Center of Regenerative Medicine and Stem Cell Research, Intellectual and Developmental Disabilities Research Center, University of California, Los Angeles, Los Angeles, CA, United States; ^3^Department of Health, Kinesiology, and Sport, University of South Alabama, Mobile, AL, United States; ^4^Department of Psychology, University of South Alabama, Mobile, AL, United States; ^5^Department of Physician Assistant Studies, University of South Alabama, Mobile, AL, United States; ^6^College of Medicine, University of South Alabama, Mobile, AL, United States; ^7^Department of Internal Medicine, College of Medicine, University of South Alabama, Mobile, AL, United States

**Keywords:** neurovascular dysfunction, health disparities, Alzheimer’s disease, Alabama (United States), dementia

## Abstract

Alzheimer’s disease and related dementias (ADRD) are an expanding worldwide crisis. In the absence of scientific breakthroughs, the global prevalence of ADRD will continue to increase as more people are living longer. Racial or ethnic minority groups have an increased risk and incidence of ADRD and have often been neglected by the scientific research community. There is mounting evidence that vascular insults in the brain can initiate a series of biological events leading to neurodegeneration, cognitive impairment, and ADRD. We are a group of researchers interested in developing and expanding ADRD research, with an emphasis on vascular contributions to dementia, to serve our local diverse community. Toward this goal, the primary objective of this review was to investigate and better understand health disparities in Alabama and the contributions of the social determinants of health to those disparities, particularly in the context of vascular dysfunction in ADRD. Here, we explain the neurovascular dysfunction associated with Alzheimer’s disease (AD) as well as the intrinsic and extrinsic risk factors contributing to dysfunction of the neurovascular unit (NVU). Next, we ascertain ethnoregional health disparities of individuals living in Alabama, as well as relevant vascular risk factors linked to AD. We also discuss current pharmaceutical and non-pharmaceutical treatment options for neurovascular dysfunction, mild cognitive impairment (MCI) and AD, including relevant studies and ongoing clinical trials. Overall, individuals in Alabama are adversely affected by social and structural determinants of health leading to health disparities, driven by rurality, ethnic minority status, and lower socioeconomic status (SES). In general, these communities have limited access to healthcare and healthy food and other amenities resulting in decreased opportunities for early diagnosis of and pharmaceutical treatments for ADRD. Although this review is focused on the current state of health disparities of ADRD patients in Alabama, future studies must include diversity of race, ethnicity, and region to best be able to treat all individuals affected by ADRD.

## Introduction

ADRD remain a global health crisis for all affected individuals including patients with ADRD, individuals at risk, and caregivers such as friends and family. For over a century, ADRD research has made significant efforts to cure, treat, or prevent ADRD. However, there is a clear lack of research focusing on regional, racial, and ethnic disparities in ADRD, particularly in Southern, rural and poor states like Alabama. It is estimated that over 94,000 people in Alabama have AD (Alzheimer’s Association, 2022) and AD is the sixth leading cause of death of people in Alabama (Alabama, 2021). African Americans are more likely to develop AD than White Americans (Blum et al., 2018; Alzheimer’s Association, 2022). According to the US census as of 2021, African Americans account for 13.4% of the American population, but in Alabama 26.8% of the over 5 million people are African American (Census, 2022), presenting a greater risk and prevalence of ADRD in Alabama. In addition, more than a million Alabamians live in rural areas, where there are even more significant health disparities (Rural Health Information Hub, 2022). This combination of high-risk populations presents an urgent need for research focusing on ADRD in Alabama and other regions with similar demographics.

The neuropathologies found in the post-mortem brains of ADRD patients are complex and multifactorial. A number of amyloid isotypes accumulate in the brains of these patients including amyloid-β (Aβ) plaques, neurofibrillary tau tangles (NFTs), Lewy body α-synuclein pathologies, and transactive response DNA and RNA binding protein 43 kDa aggregates (Nelson et al., 2019; Robinson et al., 2021; Uemura et al., 2022). These neuropathologies are often associated with neurovascular abnormalities including large macroscopic, lacunar and microscopic infarcts, hemorrhages, and vessel pathologies including cerebral amyloid angiopathy, intracranial atherosclerosis and arteriolosclerosis. For example, in the Religious Order Study and Rush Memory and Aging Project cohort, ∼87% of probable AD diagnosed subjects had abnormal vascular neuropathologies. Approximately 74% of these subjects also had traditional AD and/or other neurodegenerative neuropathologies (Kapasi et al., 2017). This work and others described below support that neurovascular dysfunction occurs more often than not in ADRD.

Neurovascular dysfunction in ADRD can be partially attributed to cardiovascular deficits (Shabir et al., 2018). Neurovascular uncoupling, an early event in AD, leads to dysregulation of cerebral blood flow (CBF) and the NVU and is a major contributor to AD progression (Iadecola, 2004, 2013; Zlokovic, 2011; Montagne et al., 2015; Sweeney et al., 2015, 2019; Zhao et al., 2015; Arvanitakis et al., 2016; Iturria-Medina et al., 2016; Nelson et al., 2016; Kisler et al., 2017a; Nation et al., 2019). Blood-brain barrier (BBB) breakdown as well as pericyte injury and loss are also hallmark findings in AD, leading to chronic neuroinflammation, gliosis, Aβ deposition, and tau hyperphosphorylation (Collins-Praino and Corrigan, 2017; Perea et al., 2018). The aforementioned clinical findings in AD may be partially mitigated by education and awareness of several extrinsic vascular risk factors linked to AD. The most common genetic risk factor for AD is apolipoprotein ε4 (*APOE4*) carriage (Mahley et al., 2006). Studies have suggested that *APOE4* mice (Bell et al., 2012) and humans (Montagne et al., 2020a) have increased BBB breakdown that corresponds with cognitive decline. Furthermore, APOE functions to transport lipids (e.g., cholesterol) in the bloodstream (Di Battista et al., 2016). Previous studies indicate that *APOE4* carriage disrupts brain cells’ ability to metabolize lipids (Dupuy et al., 2001; Huang and Mahley, 2014). Metabolism deficiencies may be the cause of gut dysbiosis seen in AD cases, which contributes to increased proinflammatory cytokine levels and systemic inflammation (Al Bander et al., 2020). Furthermore, systemic infections, such as pneumonia, lead to peripheral generation of amyloids (e.g., Aβ and tau) and incident dementia that may ultimately contribute to ADRD (Nelson, 2022). Stress and anxiety also contribute to AD progression through consequences in subsequent behavioral and physiological changes (Dimsdale, 2008). Diet and lifestyle differences have also been identified as a risk factor for AD. Use of alcohol and recreational drugs is linked to ADRD diagnosis and cognitive impairment. These identified risk factors not only contribute to AD progression, but related cardiovascular and pulmonary diseases as well. For this reason, cardiovascular and pulmonary diseases have been linked to dementia via disruption of the BBB and neuroinflammation. However, it is important to note that vascular risk factors that occur in midlife may be temporally uncoupled with cognitive dysfunction, suggesting that aging is a contributing factor to neurovascular dysfunction in ADRD.

Although there is no cure for ADRD, several medications may be prescribed to temporarily alleviate symptoms through decreasing hypertension, protecting neurovasculature, or correcting gut dysbiosis (Ahmed, 2005; Stirban et al., 2006; Raj et al., 2018; Williamson et al., 2019; Nasrallah et al., 2021). Non-pharmaceutical treatments may also be viable options to slow cognitive decline in AD patients, also commonly through the correction of gut dysbiosis. Physical activity has been shown to decrease the risk of AD and slow cognitive decline, even in *APOE4* carriers (Allard et al., 2017). However, the South has the highest prevalence of physical inactivity in the United States (Centers for Disease Control and Prevention, 2021). Treatment of mood disorders has also been shown to protect vascular health through prevention of adopting unhealthy behaviors such as smoking and physical inactivity (Abed et al., 2014). Other non-pharmaceutical and alternative treatments for AD such as hormone replacement therapy and oxygen therapy have also been suggested to treat patients with AD, but their efficacies are still unclear (Smith et al., 2010; Harch and Fogarty, 2018; Guo et al., 2020; Song et al., 2020; Shapira et al., 2021; Somaa, 2021).

Every individual has a unique social determinants of health profile and many individuals living in Alabama suffer from inequities in health and health care. Social determinants of health are defined as, “the conditions in the environments where people are born, live, learn, work, play, worship, and age that affect a wide range of health, functioning, and quality-of-life outcomes and risks” (Healthy People, 2022). Health disparities are usually manifested in groups that are disadvantaged. Individuals and groups may be disadvantaged due to their race or ethnicity, sex, sexual identity, age, disability, SES, cognitive abilities, and geography (Foundation Health Measures Archive, 2020). In most of the US and in Alabama specifically, SES and race/ethnicity are the areas of disadvantage that have the greatest impact on health disparities (Arrieta et al., 2008).

Although there are numerous potential treatments to slow cognitive decline and vascular dysfunction progression in AD, many social and structural determinants of health in Alabama make it difficult to access these treatments or even diagnose ADRD. Many regions within Alabama suffer from low SES creating the challenge to adopt healthy eating and exercise habits that might prevent or delay onset of ADRD. Diagnosis of dementia tends to be earlier in individuals with high SES, when interventions may have an impact, than people with low SES (Cha et al., 2021; Petersen et al., 2021). Many areas in Alabama, both urban and rural, have limited access to healthcare and healthy food, making it more difficult to initiate crucial lifestyle changes to slow AD progression. The large rural population of Alabama further adds to the increased prevalence of AD. Taken together, it is clear that this region presents numerous risk factors of ADRD as well as substantial barriers preventing the mitigation of these risks. Reports are desperately needed to highlight the urgency of developing preventative strategies targeted at ADRD, especially in states with an ethnic and racial makeup like Alabama. Low SES and health disparities exist in global communities contributing to ADRD worldwide. Therefore, in this review, we present the current social and structural determinants of health that lead to health disparities contributing to neurovascular dysfunction and ADRD in Alabama and beyond.

## Neurovascular Dysfunction in Alzheimer’s Disease and Dementia

### Neurovascular Uncoupling and Cerebral Blood Flow Reductions

**FIGURE 1:**
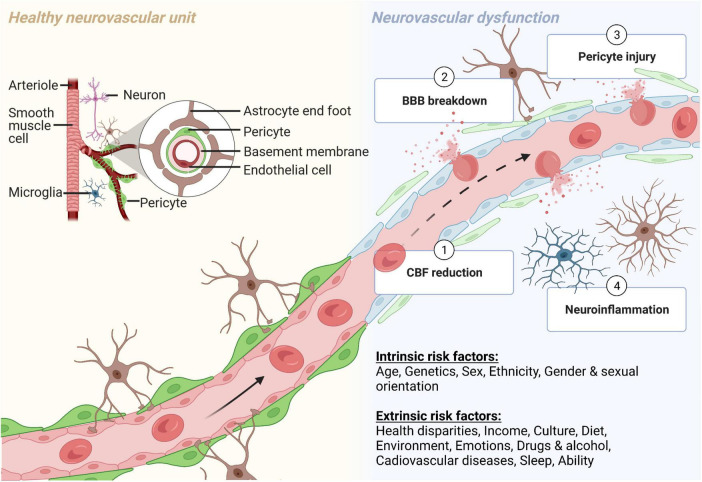
The neurovascular unit (NVU) during normal physiological and pathological conditions. The NVU is comprised of many cell types. The blood-brain barrier (BBB) is formed by endothelial cells, which at the capillary level, are supported by pericytes. Astrocytic endfeet provide additional support to the BBB. Other cellular components of the NVU include microglia and neurons (*yellow, left side*). (1) Cerebral blood flow (CBF) reductions, (2) BBB breakdown, (3) pericyte injury, and (4) neuroinflammation all contribute to neurovascular dysfunction in many neurodegenerative diseases and disorders, including Alzheimer’s disease (*blue, right side*). Both intrinsic and extrinsic factors can contribute to neurovascular dysfunction. Created with BioRender.com.

The brain is metabolically active and requires 20% of the body’s oxygen and glucose for proper functioning (Iadecola, 2013). The NVU consists of vascular cells (e.g., endothelial cells, pericytes, and vascular smooth muscle cells), glia (e.g., astrocytes, microglia, and oligodendrocytes), and neurons (Nelson et al., 2016; Sweeney et al., 2019) (Figure 1). Neurovascular coupling refers to the communication and connection between the vasculature and brain cells. The NVU regulates CBF through neurovascular coupling to assure that brain energy needs are met (Kisler et al., 2017a). There is growing appreciation and strong evidence that neurovascular uncoupling, CBF reductions and dysregulation, and breakdown of the BBB, including the loss of pericytes, are early events in the AD pathophysiological cascade (Iadecola, 2004, 2013; Zlokovic, 2011; Montagne et al., 2015, 2020a; Sweeney et al., 2015, 2019; Arvanitakis et al., 2016; Iturria-Medina et al., 2016; Nelson et al., 2016; Kisler et al., 2017a) (Figure 1). In a clinical study, African Americans were found to have lower intracranial arterial blood flow than White people which was associated with higher fasting glucose and triglyceride levels (Clark et al., 2019).

### Blood-Brain Barrier Breakdown

The BBB has been referred to as the gatekeeper of the brain. Increased adherens and tight junctions in the BBB prevent uninhibited entry of blood-derived products, toxins, and molecules from entering the brain (Zlokovic, 2011; Zhao et al., 2015; Nelson et al., 2016). Unlike peripheral vessels, the BBB has a controlled specialized substrate-specific transport system (Zlokovic, 2011; Zhao et al., 2015; Nelson et al., 2016).

BBB breakdown in AD has been detected by immunohistochemistry (Salloway et al., 2002; Bailey et al., 2004; Wu et al., 2005; Baloyannis and Baloyannis, 2012; Sengillo et al., 2013; Halliday et al., 2016), fluid biomarker assessment of albumin quotient, plasminogen and fibrinogen (Craig-Schapiro et al., 2011; Montagne et al., 2015; Sweeney et al., 2015), and magnetic resonance imaging neuroimaging sequences of perivascular hemosiderin deposits/microbleeds (Goos et al., 2009; Brundel et al., 2012; Uetani et al., 2013; Heringa et al., 2014; Olazarán et al., 2014; Yates et al., 2014; Zonneveld et al., 2014; Shams et al., 2015; Poliakova et al., 2016). More recent studies have used dynamic contrast enhanced magnetic resonance imaging to quantify BBB permeability and found increased BBB breakdown in individuals with normal aging (Montagne et al., 2015; Verheggen et al., 2020), MCI (Montagne et al., 2015, 2020a; Nation et al., 2019; Rensma et al., 2020), and early AD (van de Haar et al., 2016, 2017a,b).

### Pericyte Injury and Loss

With endothelial cells being the gatekeepers of the brain, pericytes function as the padlock, providing a second layer of protection to ensure the gate regulated by the BBB is fortified. Pericytes are critical support cells of the BBB and have other key functions including angiogenesis, clearance of toxic metabolites (Ma et al., 2018), and regulating capillary hemodynamic responses (Kisler et al., 2017a,b; Nelson et al., 2020). Pericyte loss has been demonstrated using electron microscopy of AD cortex (Farkas and Luiten, 2001; Baloyannis and Baloyannis, 2012) and by decreased levels of pericyte marker platelet derived growth factor receptor β (PDGFRβ) in the precuneus and underlying white matter (Miners et al., 2018). Through immunohistochemical analysis, it was shown that pericyte number and coverage of brain capillaries were reduced in the AD cortex and hippocampus when compared to control brains (Sengillo et al., 2013), and this loss was accelerated in *APOE4* carriers (Halliday et al., 2016). Pericyte injury marker soluble PDGFRβ has also been found to be elevated in cerebrospinal fluid (CSF) in MCI and early AD (Montagne et al., 2015, 2020a; Miners et al., 2019; Nation et al., 2019; Wang et al., 2022). No studies to date, that we could identify, have assessed BBB breakdown or pericyte injury in diverse populations with MCI or ADRD.

### Neuroinflammation

Neuroinflammation may be triggered by injury, infection, stress, or aging (DiSabato and Sheridan, 2021), and short-term neuroinflammation generally leads to improved patient outcome (Hopper et al., 2012). However, neuroinflammation can be prolonged and may become detrimental when the stimulus persists, leading to neuronal dysfunction, injury, or deficit (Streit et al., 2004; Delgado et al., 2021). Specific to AD, neuroinflammation has been correlated with increased levels of proinflammatory cytokines such as tumor necrosis factor-α (TNF-α) and interleukin (IL)-6 (Strauss et al., 1992; Chang et al., 2017) in both the brain and blood. Another study comparing the release and presence of microvessel-associated cytokines between AD and control brain microvessels found increased levels of IL-1β, IL-6, and TNF-α in AD brains (Grammas and Ovase, 2001). IL-1β has been shown to activate certain kinases that promote tau hyperphosphorylation (Collins-Praino and Corrigan, 2017). Proinflammatory cytokines have also been shown to increase Aβ production via upregulation of beta-secretase 1 (BACE1), the key enzyme that initiates production of Aβ (Vassar, 2004).

Neuroinflammation can be beneficial or detrimental and is mediated by astrocytes and microglia (Fakhoury, 2018). Microglia are the primary immune cells of the central nervous system (CNS), act as resident macrophages (DiSabato and Sheridan, 2021) and are important for synapse integrity and learning and memory (Wolf et al., 2017). Microglia are activated by Aβ plaques as well as hyperphosphorylated tau (Perea et al., 2018). Although microglia initially provide neuroprotection through clearance and degradation of Aβ, prolonged activation leads to hypersecretion of proinflammatory cytokines (Shastri et al., 2013) and pronounced proliferation of microglia and inflammatory markers (Varnum and Ikezu, 2012). Preclinical studies from AD models suggest specialized proresolving mediator (SPM) deficiencies and dysregulation due to imbalance between proinflammatory cytokines and SPM or SPM synthesis interruption (Ponce et al., 2022). Microglia also communicate with astrocytes, specialized glial cells that are the most abundant cells in the CNS (Shastri et al., 2013). IL-1β, a cytokine that functions in astrocyte proliferation and astrogliosis, has been shown to be present in 30x as many glial cells in AD brains compared to age-matched controls (Griffin et al., 1989; Grammas and Ovase, 2001).

Aβ in both its plaque and soluble form activates the pathway of microglia priming, leading to release of reactive oxygen species. Microglial priming in AD patients is exceptionally detrimental because the cytokines and chemokines released during this process fuels a positive feedback mechanism or neuroinflammation inducing astrogliosis, Aβ deposition, as well as further release of proinflammatory cytokines (Ho et al., 2005; Delgado et al., 2021). Further studies regarding neuroinflammation should include various races and ethnicities to reveal potential variance in neuroinflammatory patterns in diverse populations.

## Vascular Risk Factors Linked to Alzheimer’s Disease

### Race and Ethnicity

An analysis of 2014 Medicare and US Census Bureau data found that ADRD were most prevalent in Black Americans and Hispanic adults over the age of 65 years. The rates were 14.7% for African Americans, followed by 12.9% for Hispanic adults, 11.3% for non-Hispanic White people, 10.5% for American Indian and Alaska Natives, and 10.1% for Asian and Pacific Islanders (Matthews et al., 2019).

A study focusing on plasma metabolites in AD subjects across multiple races and ethnicities showed that amino acid metabolism was altered in African Americans, non-Hispanic White people, and Caribbean Hispanics adults. Fatty acid metabolism was altered in African Americans and non-Hispanic White people. African Americans also had altered glycolytic metabolism (Vardarajan et al., 2020). Neurodegenerative disease panels from a study on AD in African Americans revealed that levels of soluble receptor for advanced glycation endproducts (sRAGE) were significantly elevated in African Americans (Ferguson et al., 2021). sRAGE is a receptor on the luminal side of the BBB involved in transport of Aβ into the brain and increased sRAGE levels are correlated with increased Aβ accumulation (Nelson et al., 2016). The same study that identified elevated sRAGE also found higher levels of innate immunity regulator nuclear factor kappa B and its inhibitor, nuclear factor of kappa light polypeptide gene enhancer in B-cells inhibitor-alpha, in African American AD male patients compared to white people and African American females, with a 116% increase in Fas-associated death domain protein levels for African Americans, indicating increased apoptosis (Ferguson et al., 2020). In a separate study, it was found that levels of IL-1β, monokine induced by gamma, and TNF-related apoptosis-inducing ligand were increased and levels of IL-8 and IL-3 were decreased in African Americans, independent of gender, further explaining increased incidence of apoptosis (Ferguson et al., 2021). In addition, CSF IL-9 levels were increased in African Americans with AD but not in white people with AD (Wharton et al., 2019).

The specific reasons for these differences across races and ethnicities remain unclear. Contributing to this knowledge gap is the lack of multi-ethnic studies in clinical research including studies of ADRD. There has been work to understand the barriers to enrolling participants from underrepresented groups into clinical research projects (George et al., 2014). Non-Hispanic White people were found to be the most willing to participate in ADRD research studies, while Hispanic adults, non-Hispanic Asians, and non-Hispanic Black people were 44, 46, and 64%, respectively, less willing to participate in AD prevention trials (Salazar et al., 2020). African Americans’ participation in research studies should be increased in order to better understand ADRD and other diseases that disproportionately impact them. Among African Americans, there is a long-standing mistrust in the health care system and a significant contributor to that mistrust is rooted in Alabama. The U.S. Public Health Service Syphilis Study at Tuskegee, 1932–1972 is a well-known unethical 40-year clinical study that continues to be a major barrier to the participation of Black people in research (Freimuth et al., 2001). Mistrust stems from this and other historical events and is a known barrier to research participation reinforced by discriminatory events and flaws in the healthcare system (Scharff et al., 2010). Therefore, current research in race- and ethnicity-dependent vascular risk factors for AD is also limited by small sample sizes. In addition, most multi-racial and ethnicity studies in ADRD have focused on comparing non-Hispanic White people to Black people, while inclusion of other races and ethnicities is less common. Further multi-racial studies investigating the mechanisms mediating ethnic differences in AD pathology are needed. Encouraging community engagement and involvement early in the design and development of a research project helps to build trust while also allowing the participants to share the importance of research with other members of the surrounding community (Crook et al., 2019).

### Genes

Autosomal-dominant AD (ADAD) is a form of AD caused by mutations in *PSEN1*, *PSEN2*, and *APP*. ADAD accounts for a small percentage of all AD cases and has an early age of onset (<65 years of age). The presentation and symptoms of ADAD are very similar to those seen in the more common sporadic cases of AD (Bateman et al., 2011). While sporadic AD is not primarily a genetic disease, several genes significantly increase the risk for AD development. The most common genetic risk factor of sporadic AD is *APOE4* (Mahley et al., 2006). In addition, mutations in *PICALM*, *CLU*, and *SORL1* have been implicated in AD (Zhao et al., 2015; Nelson et al., 2016; Sweeney et al., 2019). Interestingly, all the above-mentioned genes have been implicated in neurovascular dysfunction, as recently reviewed (Zhao et al., 2015; Nelson et al., 2016; Sweeney et al., 2019). Also, *ABCA7* and *TREM2* have been implicated in AD and are linked to innate immunity (Sweeney et al., 2019).

Although many genome-wide association studies (GWAS) have identified genetic risk factors in AD (Lambert et al., 2013; Naj et al., 2014; Kunkle et al., 2019; Wightman et al., 2021), these studies predominantly included Europeans or Americans of European descent. Moreover, individuals from several US states were included, but none specifically from Alabama. Recently, GWAS have been done to evaluate AD risk genes in African American populations (Logue et al., 2011, 2018; Kunkle et al., 2021), showing that African Americans and white people have variation in the top genetic risk factors for AD. Specifically, the *DRD2 Taq A1* allele reduces the D2 receptors in the brain, causing impaired cognitive function in older age, creating a magnified risk of AD (Blum et al., 2018). This A1 allele is more prevalent in African American populations. Kunkle et al., 2021, identified 8 novel loci (*TRANK1*, *FABP2*, *LARP1B*, *TSRM*, *ARAP1*, *STARD10*, *SPHK1*, and *SERPINB13*) as being significant risk factors for African Americans. This study only identified *TREM2* and *C2DAP* as being significant in both African American and White populations. *ABCA7* mutations are more prevalent in African American populations and could be a greater risk factor than *APOE4* (Stepler et al., 2022). When ATP Binding Cassette Subfamily A Member 7 (ABCA7) dysfunctions, more Aβ is produced and not cleared properly from the brain by microglia (Aikawa et al., 2018). Precision medicine is one mechanism to lessen the impact of the social determinants of health and other contributors to health disparities in the future. Understanding the genetic—environment interaction is critical if that future is to be realized. Therefore, studies seeking to identify genes conferring increased risk, likelihood of response to therapy, and prognosis for ADRD must include more diverse populations with regards to ethnicity, gender, and geography.

### Diet and Lifestyle

The Centers for Disease Control and Prevention (CDC) has recently reported that over 35% of the adult population in the US was obese in 2021, with the highest obesity rates in the Southern states (Mississippi, Louisiana, and Alabama) (CDC, 2021c). In Alabama, non-Hispanic Black adults (46.2%) encounter obesity-related health disparities more than non-Hispanic White adults (34.3%) (CDC, 2021c) with high-energy food intake and physical inactivity as main factors contributing to this problem. The 2021 county health rankings revealed that 29% of adults in Alabama had no leisure-time physical activity (Alabama, 2022). In addition, several areas in Alabama suffer from low income and limited accessibility to healthy foods (USDA, 2022). It has previously been shown that energy-dense food consumption triggers severe health conditions such as metabolic syndrome, cardiovascular disorder, neurovascular dysfunction, memory deficits, as well as AD (Thériault et al., 2016; Ting et al., 2017; Moreno-Fernández et al., 2018; Lasker et al., 2019; Bracko et al., 2020). Studies in animals fed with short-term or long-term enriched-energy diet increased the permeability of the BBB as demonstrated by an elevation of extravascular immunoglobulin G deposits and albumin content in the hippocampus (de Aquino et al., 2018; Yamamoto et al., 2019; de Paula et al., 2021). Animals fed with a high-calorie diet exhibited attenuation of tight junction proteins claudin 5 and occludin, alleviation of collagen type IV, augmented fenestration of endothelial cells, and astrogliosis (de Aquino et al., 2018; Yamamoto et al., 2019). In mice, consumption of high-salt diets were found to reduce the brain endothelial cells’ capacity to generate nitric oxide, leading to cognitive and neurovascular dysfunction (Faraco et al., 2018). These negative effects not only disrupt the structure of the NVU, but also reduce CBF, which plays a crucial role in neurovascular coupling processes (Faraco et al., 2018; Nielsen et al., 2020). Notably, both *in vivo* and clinical experiments have shown that a disruption of the BBB and neurovascular uncoupling potentially diminish cognitive performance, resulting in the development of AD and dementia (Lourenço et al., 2017; Nielsen et al., 2020; Lin et al., 2021). For these reasons, the modification of structural and social determinants of health (e.g., improved access to food, safer neighborhoods for exercise) may allow improvements in dietary behaviors and the enhancement of physical activities which are beneficial ways to decrease risk of obesity, vascular dysfunction, and AD in Alabama and other states.

### Emotions, Stress, and Anxiety

Stress has been identified as a vascular risk factor and can contribute to negative vascular outcomes through influencing behavior and physiology. A systematic review found stress to be related to behavioral factors that are known to lead to poor vascular health outcomes (Dimsdale, 2008). Stress influenced the adoption of unhealthy lifestyle habits, including smoking, eating a poor-quality diet, disordered eating, and living a sedentary lifestyle (Dimsdale, 2008). Increased levels of stress hormones, such as cortisol, have been found to impair cerebrovascular function (Münzel et al., 2018). Chronic mental stress was found to be a risk factor for metabolic syndrome and for the development of hypertension (Esler et al., 2008). Stress facilitates the progression of AD and exacerbates symptom severity (Justice, 2018). Additionally, AD patients exhibit increased levels of stress hormones (Csernansky et al., 2006). Stress is a psychological factor that impacts behavioral and physiological functioning, consequentially impacting vascular health and increasing the risk of AD.

Anxiety is an independent cardiovascular disease risk factor (Shen et al., 2008). Additionally, it has been linked to adverse cardiovascular outcomes such as autonomic dysfunction, inflammation, and endothelial dysfunction (Celano et al., 2016). A meta-analysis of 46 studies found anxiety to be associated with several vascular events such as a 71% higher risk of stroke, a 41% higher risk of cardiovascular mortality and coronary heart disease, and a 35% higher risk of heart failure (Emdin et al., 2016). In a study examining neuronal activity and blood supply in individuals with generalized anxiety disorder (GAD), patients with GAD displayed a decrease in neurovascular coupling and alteration in CBF (Chen et al., 2021).

Allostatic load is the cumulative burden of chronic stress and life events (Guidi et al., 2021). Black Americans have a greater allostatic load than White Americans, which was shown to be associated with poorer physical and mental health outcomes (Guidi et al., 2021). Living with the negative impacts of racism is a significant stressor on Black Americans and other minorities. A study using multinomial logistic regression to quantify experiences of racism and subjective cognitive function found that Black women subjected to institutional racism had worsened subjective cognitive function that was in part mediated by depression and insomnia (Coogan et al., 2020). Another study found that higher perceived stress in older Black Americans was associated with faster declines in global cognition, especially episodic memory and visuospatial ability (Turner et al., 2017). Stressful life events were reported more frequently in Black Americans than White people and were associated with age-related cognitive decline (Zuelsdorff et al., 2020). Stress in Black Americans has been linked to elevated Aβ and tau CSF biomarkers (Garrett et al., 2019; Trammell et al., 2020). Modifying environmental (e.g., larger living space), sociocultural (e.g., larger social network size), behavioral (e.g., more purpose in life), and biological (e.g., higher global cognition) levels was associated with a lower odds of having higher levels of perceived stress (Glover et al., 2021). This suggests that improving social determinants of health, including those affecting Black Alabamians, may help alleviate stress-associated cognitive decline and ADRD.

### Drugs and Alcohol

There is a positive association between heavy drinking (as defined by the World Health Organization) and diagnosis with dementia or cognitive impairment (Schwarzinger et al., 2018). A clinical review in 2018 described that chronic heavy alcohol consumers had an increased risk of dementia and cognitive impairment (Topiwala and Ebmeier, 2018). For example patients with chronic alcoholism were discussed to have a frontal lobe more vulnerable to dementia (Topiwala and Ebmeier, 2018). It is already known that frontal cortex and hippocampus size and function are significantly altered with alcoholism while white matter recovery is seen in cases upon abstinence from alcohol (Alvarez et al., 1989; Bengoechea and Gonzalo, 1991; Kril and Halliday, 1999). Although alcohol has been identified as a risk factor for AD, a statistically significant association between light use of alcohol and lower risk of cognitive decline and dementia has also been found (Yan et al., 2021). However, studies presented are limited by obvious ethical obstacles and inherent biases. That is, alcohol use is always self-reported, studies often lack controls for confounding variables, and there are few studies that examine interactions between other risk factors of AD such as drug use and genetics.

Smoking tobacco has been identified as a modifiable risk factor for AD (Etgen et al., 2011). In 2021, it was reported that approximately 18.5% of adults in Alabama smoked cigarettes (Explore Smoking in Alabama, 2021 Annual Report), whereas the national prevalence is only 12.5%. A longitudinal study suggested that smokers should be encouraged to quit because continual smokers have an increased risk of overall dementia (Choi et al., 2018). The same study also showed that long-term quitters as well as individuals who have never smoked had a decreased risk of developing AD as well as vascular dementia when compared to continual smokers (Choi et al., 2018). It is hypothesized that a combination of smoking-induced oxidative stress and the brain’s high susceptibility to oxidative stress contributes to neuroinflammation via release of proinflammatory cytokines and gliosis (Voloboueva and Giffard, 2011). Oxidative stress is known to be a consequence of Aβ- or tau-based neuropathies (Praticò et al., 2002; Sutherland et al., 2013). However, an *in vitro* study reported that oxidative stress stimulates BACE1 transcription which subsequently promotes Aβ production (Tamagno et al., 2008).

Certain prescription drug use has also been identified as a risk factor for AD. Benzodiazepines have been shown to induce memory deficit states through targeting of gamma-Aminobutyric acid (GABA), and it is proposed that the α5 subunit of GABA is most affected as it controls cognitive functions (Ettcheto et al., 2019).

Recreational use of marijuana has also been identified as a risk factor for AD, although related studies present mixed results. For instance, it has been demonstrated that marijuana users exhibited a lower average cerebral perfusion and decreased right hippocampal perfusion (Amen et al., 2017). However, the active ingredient in marijuana (i.e., THC) is a competitive inhibitor of acetylcholinesterase, a critical region involved in the formation of Aβ (Eubanks et al., 2006). Therefore, it is shown that low doses of marijuana may have therapeutic effects on AD (Cao et al., 2014).

### Sex, Gender, and Sexuality

Two-thirds of the Americans affected by AD are women (Alzheimer’s Association, 2022). Originally, this was attributed to women’s longer lifespan since AD development increases with age (Mielke et al., 2014). However, further research suggests that there may be other factors contributing to this sex difference than solely longevity (Snyder et al., 2016). One study examining *APOE4*-by-sex interaction as a risk of converting from healthy aging to MCI/AD suggested that the effect of *APOE4* carriage was stronger in women (Altmann et al., 2014). The same study also showed more elevated Aβ in the CSF of women compared to men (Altmann et al., 2014). There is still an evident gap in knowledge regarding sex differences in AD onset and progression and more studies should focus on gender- and sex-based disparities of ADRD risk factors.

Approximately 3.1% of adults in Alabama identify as lesbian, gay, bisexual, transgender, queer, or questioning (LGBTQ) (Williams Institute, 2022). Individuals in the LGBTQ community experience various health disparities and exhibit a greater risk for dementia than their straight, cisgender counterparts. For example, researchers found increased reports of subjective cognitive decline in LGBTQ individuals compared to straight, cisgender participants (Flatt et al., 2021). Human immunodeficiency virus, acquired immunodeficiency syndrome (HIV/AIDS) is most prevalent within the LGBTQ community, and is also linked to AD (Davis, 2013). BBB breakdown plays a major role in HIV-associated dementia by allowing HIV-1-infected monocyte-macrophages to traverse the BBB and enter the brain (Strazza et al., 2011; Sweeney et al., 2019). Once HIV accesses the brain, it negatively influences cognitive function by eventually leading to Aβ plaque deposition and/or NFTs (Canet et al., 2018).

LGBTQ individuals are less likely to seek needed medical care due to lifetime experiences of discrimination and victimization (Quinn et al., 2015). LGBTQ Americans are also less likely to develop support systems through marriage or having children and are twice as likely to live alone (Zelle and Arms, 2015; Goldsen et al., 2017). This lack of support may have negative effects on mental health and lead to increased stress, a known vascular risk factor for AD (Caruso et al., 2018). One notable research study conducted by multiple universities is Research Inclusion Supports Equity (RISE). The RISE study functions to ensure that LGBTQ adults are properly represented in ADRD research.^1^ More studies must bridge the gap between healthcare and research of LGBTQ individuals and the heterosexual population by including LGBTQ demographics in health-related research.

### Pathological Diseases

#### Systemic Inflammation and Gut Dysbiosis

Systemic inflammation is an important mechanism linked to vascular impairment and AD (Takeda et al., 2013; Elwood et al., 2017). Previous studies have demonstrated a high level of proinflammatory markers in the periphery in AD subjects (Cattaneo et al., 2017). Multiple studies have revealed the association between peripheral inflammation and various diseases including gut dysbiosis, metabolic disease, diabetes, and cardiovascular disease (Cox et al., 2015; García et al., 2017; Brandsma et al., 2019; Al Bander et al., 2020).

Gut dysbiosis is an imbalance of intestinal microbial composition and is related to dietary alteration or pathological conditions, including inflammation and oxidative stress (Proctor et al., 2017; Dumitrescu et al., 2018; Al Bander et al., 2020). A reduction of *Bacteroidetes* and an enhancement of *Actinobacteria* was detected in AD patients (Zhuang et al., 2018). However, an increase of *Bacteroidetes* along with the reduction of *Firmicutes* and *Bifidobacterium* was also measured in fecal samples of AD participants (Vogt et al., 2017). All changes of gut components related to the production of proinflammatory markers in the blood subsequently caused systemic inflammation (Al Bander et al., 2020). In addition, the mentioned differences of gut microbiomes is linked to the deterioration of intestinal integrity, which leads to the leakage of lipopolysaccharides as well as gram-negative bacteria into blood circulation (Thiennimitr et al., 2018; Salguero et al., 2019; Marizzoni et al., 2020). These microbiome differences are also associated with changes of short-chain fatty acids levels in blood, which contribute to peripheral inflammation (Marizzoni et al., 2020). Systemic inflammation or gut dysbiosis increases vascular permeability as demonstrated by an elevation of albumin in brain parenchyma (Takeda et al., 2013) and attenuates the level of tight junction protein expression in brain endothelial cells (Hu et al., 2020; Wen et al., 2020). These effects caused by systemic inflammation and gut dysbiosis ultimately contribute to cognitive decline in ADRD.

Gut microbiota alteration and/or systemic infection may disproportionately affect ethnic minorities, though the influence of race and ethnicity on gut composition remains to be fully discovered. One study observed variation in the phyla of microbiota, including *Actinobacteria, Firmicutes, Proteobacteria, Bacteroidetes*, and *Verrucomicrobia* across ethnicities (Brooks et al., 2018). There was also significant variation of gut microbiota across ethnicities as determined by Shannon’s alpha diversity index from the American Gut Project which ranked Hispanic adults greater than white people, followed by Asians, then African Americans (Brooks et al., 2018). Furthermore, the relationship of patients with infectious diseases, such as ulcerative colitis, and ethnic disparities was shown in a previous study (Castaneda et al., 2017). Taken together, ethnicity and race are factors contributing to gut dysbiosis and inflammation in US population as well as in Alabama. Nevertheless, the link between racial and ethnic disparities and gut dysbiosis as it is associated with neurovascular impairment and cognitive decline in AD should be further studied.

#### Metabolic Syndrome and Vascular Dysfunction

**FIGURE 2:**
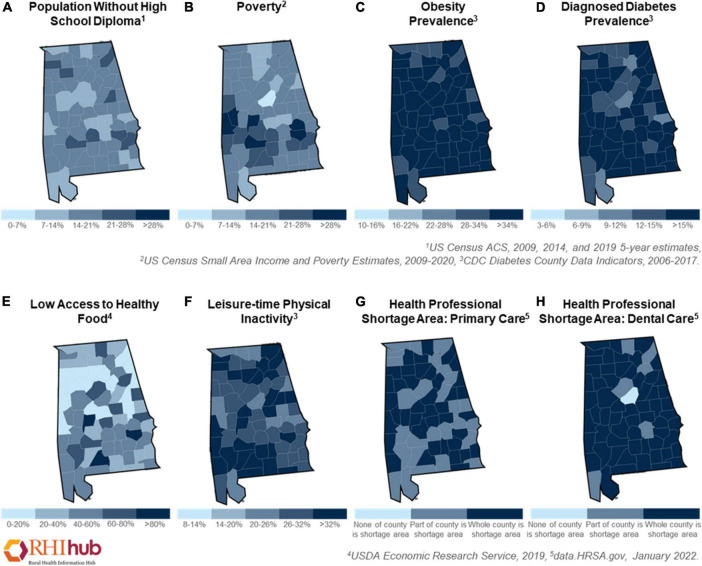
Health disparities in Alabama. The disparities prevalence of Alabamians in 2006–2022 as shown by **(A)** population without school diploma, **(B)** poverty, **(C)** the prevalence of obesity, **(D)** the prevalence of diagnosed diabetes, **(E)** low access to healthy food, **(F)** leisure-time physical inactivity, **(G)** health professional shortage area in primary care, and **(H)** health professional shortage area in dental care. These figures were generated using Rural Health Information Hub (RHIhub, www.ruralhealthinfo.org).

Metabolic syndrome is a group of metabolic risk factors characterized by a large waist circumference, abnormality of lipid profiles (e.g., high triglycerides and low high-density lipoprotein cholesterol levels), hypertension, and hyperglycemia (NHLBI, 2022). Being overweight or obese as a consequence of poor diet is the main cause of metabolic syndrome (NHLBI, 2022). Previous studies have demonstrated that a high caloric intake not only leads to hyperlipidemia but also triggers insulin resistance, both of which are linked to pre-diabetes and type 2 diabetes (Lozano et al., 2016; Saiyasit et al., 2020; Kumar et al., 2021). The national diabetes report from the CDC indicated that approximately 88 million adults in the United States had pre-diabetes and 26.8 million were diagnosed with diabetes (CDC, 2022c). In Alabama, 14.8% of adults currently have diabetes, which was shown to affect more African Americans (18.1%) than White people (14.1%) Americans in 2020 (Explore Diabetes in Alabama, 2021 Annual Report). Additionally, Alabamians with less education (Figure 2A) and low income (Figure 2B) were more susceptible to diabetes (Explore Diabetes in Alabama, 2021 Annual Report). Several studies have shown an association between diet-induced obesity or diabetes with vascular dysfunction (Chang et al., 2014; de Paula et al., 2021; Li et al., 2021). Excessive caloric consumption stimulates peripheral immune cells, induces the generation of proinflammatory cytokines and increases oxidative stress, which have all been linked to BBB dysfunction (Salameh et al., 2019). In a recent study in obese Zucker rats, researchers observed BBB disruption (e.g., increased aquaporin-4 and reduced glucose transporter 1) in the frontal cortex and hippocampus, which was associated with cognitive impairment (Tomassoni et al., 2020). BBB breakdown was also found in a streptozotocin-induced diabetes model that had reduced levels of tight junction proteins (e.g., occludin, and zonula occludens-1), and increased protein levels of cell adhesion molecules (e.g., intercellular adhesion molecule 1, and vascular adhesion protein 1) (Aggarwal et al., 2018). Importantly, BBB leakage leads to the activation of glial cells, resulting in the release of proinflammatory cytokines in the brain, causing neuroinflammation and cognitive decline (de Aquino et al., 2018; Salameh et al., 2019; de Paula et al., 2021). Notably, a large amount of Aβ accumulation was detected in the brain of a high-fat diet-induced obese model (Busquets et al., 2017). All the aforementioned studies support the theory that high-caloric consumption causes metabolic disturbance and disrupts neurovascular function, which enhances the risk of ADRD. As mentioned above, many Alabamians struggle with obesity (Figure 2C) and diabetes (Figure 2D), prompted by a high-calorie diet and/or reduced access to healthy food (Figure 2E). For these reasons, metabolic disturbances in Alabama may contribute to the increased risk for neurovascular dysfunction and ultimately progression of AD.

#### Cardiovascular Disease

Another important factor for neurovascular disruption is cardiovascular impairment (Aires et al., 2020; Vanherle et al., 2020). Based on the data in 2018, heart disease (mostly coronary heart disease) is the most common cause of death in the US, as well as in the state of Alabama (Alabama, 2021; Heart Disease and Stroke Statistics, 2021 Update). In 2020, the CDC reported the number of people in Alabama who died with heart disease: 14,739 per 100,000 individuals (Stats of the States Heart Disease Mortality, 2021). Cholesterol deposition inside the arterial wall, also known as atherosclerosis, is a vascular disease that underlies major ischemic events such as myocardial infarction and stroke (Atherosclerosis, 2022). It has been shown that there is a relationship between a score of vascular risk factors and AD risk factors in middle aged adults (Lockhart et al., 2021). A lower score of cardiovascular risk factors, aging, and incidence of dementia (CAIDE) was found in White individuals when compared with African Americans, resulting in a decreased risk for AD (Lockhart et al., 2021). A higher CAIDE score is also linked to increased Aβ deposition (Lockhart et al., 2021). Therefore, the impact of the social determinants of health in ethnic minority communities, particularly African Americans, leads to higher risk of vascular disease and AD.

Notably, the correlation of atherosclerosis, cardiovascular disease, and *APOE4* was reported (Bennet et al., 2007; Granér et al., 2008; Duong et al., 2021). *APOE4* expression was highly correlated to neurovascular impairment, stroke, and AD (Montagne et al., 2020b; Pendlebury et al., 2020). In addition, mice with targeted replacement of *ApoE* with human *APOE4* displayed a reduction of resting CBF and a lower density of brain vascular structures such as endothelial cells and pericytes (Koizumi et al., 2018). *APOE4* carriers exhibited degradation of BBB structures via several pathways, including pericyte degeneration, reduction in low density lipoprotein receptor-related protein 1 expression, increased proinflammatory cytokines, and elevated apoptosis. *APOE4* carriers also exhibited synaptic dysfunction, hyperphosphorylated tau, and increased Aβ levels (Halliday et al., 2016; Zhao et al., 2020). Furthermore, an association of increased BBB permeability and elevated levels of the pericyte injury marker, soluble PDGFRβ, in CSF and cognitive impairment was found in *APOE4* carrier participants (Montagne et al., 2020a). Therefore, cardiovascular disease not only causes systemic pathologies, but can also progress into cerebrovascular dysfunction and eventually AD, including in individuals living in Alabama.

#### Pulmonary Diseases

Human lungs are constantly exposed to airborne microbes, pollutants, and small particulates, all of which can directly impact their function, and secondarily the health of the brain. Specific to the lung-brain axis, emerging evidence indicates that damage to the lung can lead to cognitive impairment (Ely et al., 2004). The intricate lung-brain link has been implicated in lung damage associated with mechanical ventilation, bacterial and viral pneumonia, and air pollution.

Clinical studies have shown that many patients in the intensive care unit (ICU) suffer from the rapid onset of delirium during their ICU stay, which may transition into prolonged cognitive sequelae even after the patients recover from the critical illness and are released from the ICU (Girard et al., 2010). In fact, a hospital stay itself can harm cognition, and in the ICU settings, animal studies have implicated that mechanical ventilation can increase peripheral inflammation that results in neuroinflammation and impairs brain function (Reade and Finfer, 2014; Lahiri et al., 2019). While the mechanisms underlying clinical delirium and/or the consequent impairment to the brain remain unclear, cognitive impairment is more rampant in infection-induced pneumonia patients, including patients that contract either community- or hospital-acquired pneumonia infections (Karhu et al., 2011; Girard et al., 2018). Although the infection-induced peripheral inflammatory response may be a common mechanism for several types of infection, emerging evidence has indicated that additional mechanisms may underlie the lung-brain axis.

It was recently discovered that lung capillary endothelium produces and releases amyloids into the surrounding milieu (Ochoa et al., 2012; Morrow et al., 2016). These amyloids include Aβ variants that possess antimicrobial activity against invading microbes (Voth et al., 2020). However, when exposed to virulent clinical bacterial strains, including bacteria such as *Pseudomonas aeruginosa*, *Klebsiella pneumoniae*, and *Staphylococcus aureus*, the amyloids produced by lung endothelium become cytotoxic. In addition to Aβ, lung endothelium also produces several tau isoforms that are also released upon bacterial infection (Balczon et al., 2017; Choi et al., 2021).

Intriguingly, virulent bacteria-elicited lung endothelial Aβ and tau gain tropism toward the brain (i.e., neurotropic), are neurotoxic to brain cells, and may cause Aβ and tau aggregation (Choi et al., 2021). Thus, a bacterial lung infection that elicits the release of endothelial Aβ and tau may induce incident dementia indirectly by triggering the senile plaques and/or NFT pathways (Lin et al., 2018; Balczon et al., 2019, 2021; Scott et al., 2020).

*Chlamydia pneumoniae* bacterium, on the other hand, has been found to invade the brain and trigger brain impairment. Indeed, along the same line of findings, viruses and viral particles (e.g., herpes viruses) found entangled in the senile plaques of AD brains, likely due to the antimicrobial activity of Aβ, have been suspected to be the cause of AD (Kumar et al., 2016; Eimer et al., 2018; Abbott, 2020). Whether these microbes could be the smoking guns that trigger AD is still under heavy debate, but it is not hard to imagine that the presence of neurotropic viruses in the brain and the consequent neuroinflammation would directly impact brain function. Studies from the recent COVID-19 pandemic show that the SARS-CoV-2 virus could also damage the brain (Kotfis et al., 2020; Mao et al., 2020). However, it appears that the virus may do so both via the lung-brain axis and via the olfactory transneuronal retrograde pathways (Barthold et al., 1990; Perlman et al., 1990; St-Jean et al., 2004). Altogether, these findings support the concept that pathogenic microorganisms cause infections generating an innate immune response and have fueled the formation of the peripheral amyloid hypothesis to cognitive impairment and AD (Nelson, 2022).

The lung-brain axis could further expand to include chemical pollutants and particulate matters < 2.5 μm (PM2.5). In addition to the risk of developing asthma, cardiovascular disease, lung disease, and premature death, emerging evidence has implicated that greater exposure to airborne pollutants is associated with an increased risk of dementia (Ehlenbach et al., 2010; Corrales-Medina et al., 2015; Peters et al., 2019). Alabama ranks 33rd among US states for air quality with a value of 7.8. This value correlates to an average exposure to PM2.5 (Explore Air Pollution in Alabama, 2021 Annual Report). This line of research has implicated that oxidative stress induced by breathing in harmful particles results in chronic respiratory and systemic inflammation, which impairs BBB integrity and triggers neuroinflammation (Moulton and Yang, 2012). There is evidence in North America, that areas with low SES have higher concentrated air pollutants (Hajat et al., 2015). Individuals with low income also tend to live nearer to sources of pollution, increasing their exposure (O’Neill et al., 2003).

## Potential Preventions and Treatments

### Pharmaceutical Treatments

**FIGURE 3:**
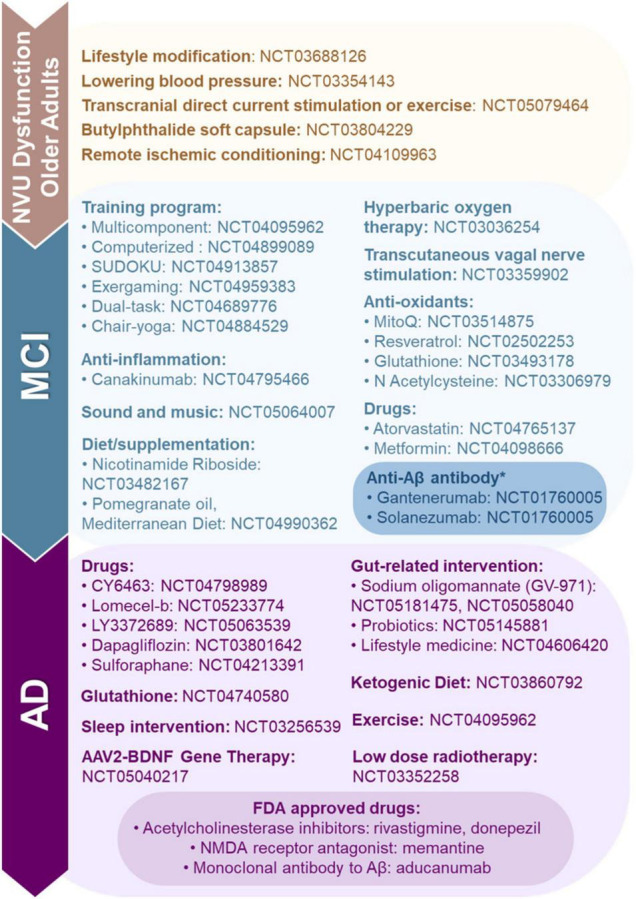
Food and Drug Administration (FDA) approved, clinical trials and preclinical studies of neurovascular unit (NVU) dysfunction, mild-cognitive impairment (MCI) and Alzheimer’s disease (AD). Here, we highlight FDA approved drugs for AD, and ongoing preclinical studies and clinical trials targeting NVU dysfunction, MCI, and AD. *Clinical trials for anti-Aβ antibodies Gantenerumab and Solanezumab are being conducted in Alabama.

There are a few drugs approved by the Food and Drug Administration (FDA) for AD patient use; these include acetylcholinesterase inhibitors (e.g., rivastigmine and donepezil), a N-methyl-d-aspartate receptor agonist (e.g., memantine), and a monoclonal antibody to Aβ (e.g., aducanumab). There are no FDA approved drugs for the treatment of neurovascular dysfunction that occurs in vascular cognitive impairment and dementia. However, there are several candidates in the pipeline which we describe below and summarize in Figure 3.

Currently, we are aware of only one interventional ADRD-related clinical trial recruiting in Alabama (NCT01760005). This study is investigating the use of anti-Aβ antibodies (e.g., Gantenerumab and Solanezumab) in patients with ADAD. These drugs have been shown to bind to Aβ aggregates and improve downstream biomarkers, but no cognitive benefits have been observed in early trials (Salloway et al., 2021).

Efforts have been made to assess the vascular and cognitive benefits of currently approved drugs. For example, lowering blood pressure was shown to reduce the risk of cognitive impairment in the Systolic Blood Pressure Intervention Trial—Memory and Cognition in Decreased Hypertension cohort (Williamson et al., 2019; Nasrallah et al., 2021) and other cohorts (Ding et al., 2020; Peters et al., 2020). The beneficial effect of anti-hypertensive classes of drugs on cognitive impairment have yielded conflicting results (De Oliveira et al., 2016; Ding et al., 2020; Peters et al., 2020).

Lomecel-b is made from medicinal signaling cells that have been isolated from bone marrow in adult donors and was recently tested in a Phase I clinical trial for AD (Brody et al., 2022). This allogenic drug functions by multimodal mechanisms of action and was able to increase anti-inflammatory (e.g., IL-10, IL-12, sIL-2Rα) and pro-vascular (e.g., VEGF, IL-4, IL-6) biomarkers in patient serum (Oliva et al., 2021).

CY6463 is a soluble guanyl cyclase stimulator that is meant to normalize the nitric oxide-cGMP signaling pathway. Deficiency in this pathway has been associated with neurovascular dysfunction (Garthwaite, 2019). This study suggests that there may be additive effects when CY6463 is administered with donepezil (Correia et al., 2021).

AD patients exhibit a decline in thiamine diphosphate-dependent enzymes, which are involved in glucose metabolism in the brain (Gibson et al., 2020). Benfotiamine is a synthetic thiamine precursor that acts on metabolic pathways, oxidative stress, and inflammation by activating transketolase which reduces advanced glycation end products production (Ahmed, 2005; Raj et al., 2018). In a Phase IIa study, Benfotiamine was determined to be safe for AD patients and lessened cognitive decline, especially in *APOE4* non-carriers (Gibson et al., 2020).

Gut microbiota play an important role in Th1/M1 microglia-predominated neuroinflammation in AD progression (Xiao et al., 2021). Sodium oligomannate (GV-971) effectively remodels gut microbiota and reduces Th1-related inflammation in the brain (Wang et al., 2020; Xiao et al., 2021). Furthermore, it was shown to improve cognitive function in AD patients in a Phase III trial (Wang X. et al., 2019; Wang et al., 2020; Xiao et al., 2021). GV-971 has been approved in China for treatment of mild to moderate AD to improve cognitive function (Syed, 2020). It has been hypothesized that the benefit of GV-971 might be due to antimicrobial and antiviral activities, specifically against herpes simplex virus type 1 (Itzhaki, 2020). Future studies should not only assess cognitive function but also examine the neurovascular benefits of GV-971 in MCI and AD patients.

### Non-pharmaceutical and Alternative Treatments

#### Diet and Supplements

While there is no standard treatment for ADRD, altering one’s diet and taking supplements may be beneficial against the cognitive decline and pathological severity of AD. Currently, multiple studies support the hypothesis that modulation of gut dysbiosis reduces negative pathologies and memory loss, delaying the severity of AD both in experimental studies and in clinical trials. Probiotic supplementations in Aβ_1–42_-induced AD rats showed improved learning and synaptic plasticity (Rezaeiasl et al., 2019). Furthermore, probiotic supplementation along with moderate-intensity interval training in AD rats enhanced the mRNA expression of hippocampal choline acetyltransferase and brain-derived neurotrophic factor (BDNF), which are essential for synaptic function (Shamsipour et al., 2021). Another experiment demonstrated that co-treatment of probiotics and selenium for 12 weeks decreased the abnormalities of metabolic profiles, reduced circulating inflammatory markers, increased antioxidant activity, and enhanced the cognitive score in AD participants (Tamtaji et al., 2019). A recent study also revealed the benefits of gut modulation on mental and stress adjustment not only in AD patients, but also in healthy older adults (Kim et al., 2021). Diet is a part of culture and may be challenging to change for individuals in the South. However, taking supplements may be an easier change to implement.

Supplementation with choline, a nutrient that plays a key role in cholinergic system and synaptic processes, reduced Aβ plaques and neuroinflammation in the hippocampus, decreased the activation of microglia, augmented synaptic proteins, and improved spatial memory in AD mice (Velazquez et al., 2019; Wang Y. et al., 2019). Furthermore, AD mice (e.g., J20) supplemented with lactoferrin, a multifunctional protein that acts as an antioxidant or anti-inflammatory, improved Aβ clearance as demonstrated by increased brain levels of ApoE and Abca1 (Abdelhamid et al., 2020). Lactoferrin supplementation not only led to a reduction of Aβ but also lowered BACE1 levels (Abdelhamid et al., 2020). Administration of ɤ-glutamylcysteine not only increased the brain antioxidant activity and anti-inflammatory expression, but also reduced lipid peroxidation, Aβ deposition, and inflammatory markers, leading to cognitive improvements in APP/PS1 mice (Liu Y. et al., 2021). Vitamin D was also shown to improve working memory at early and late stages in 5XFAD transgenic mice (Morello et al., 2018). Resveratrol, a plant-derived phytoalexin, has been shown to regulate BBB permeability and neurovascular function (Shin et al., 2015; Wei et al., 2015). Several studies showed that resveratrol administration attenuated neurovascular dysfunction (Wei et al., 2015) by enhancing antioxidants and reducing cognitive loss (Zhao et al., 2012). While the results of these experimental studies are promising, the benefits of these supplements should be tested in future clinical studies including diverse populations and regions.

A clinical study reported the benefits of dietary Omega-3 fatty acids, specifically docosahexaenoic acid and eicosapentaenoic acid, on cognition in MCI subjects (Chiu et al., 2008). Interestingly, a long-term modified ketogenic diet, a common diet for subjects with impaired brain energy metabolism, improved cognition in AD patients (Ota et al., 2019; Phillips et al., 2021).

In addition, the Mediterranean-Dietary Approach to Systolic Hypertension (DASH) diet intervention for neurodegenerative delay (MIND), which is a diet enriched with high antioxidants, has been shown to delay cognitive dysfunction associated with aging (Morris et al., 2015; de la Rubia Ortí et al., 2018; Liu X. et al., 2021). Dietary supplementation serves as a feasible and effective method to delay or protect against the progression of cognitive loss and AD. However, further studies should be done to include the effects of dietary supplementation in AD populations with diverse racial, ethnic and regional backgrounds.

#### Exercise

Individuals with higher levels of physical activity present with decreased cognitive decline and reduced risk of AD (Kramer et al., 2005, 2006; Rovio et al., 2005; Rolland et al., 2008; Lautenschlager et al., 2010; Barnes, 2015; Allard et al., 2017; Gallaway et al., 2017; Stephen et al., 2017; Barnes and Corkery, 2018; Rabin et al., 2018, 2019; Coelho-Junior et al., 2020; De la Rosa et al., 2020; Pasek et al., 2020; Sinha et al., 2020). It has been estimated that an increase of 25% in physically active adults would prevent > 230,000 cases of AD in the US (Barnes and Yaffe, 2011). This is largely based on evidence that demonstrates exercise promotes Aβ turnover (Baker et al., 2010; Liang et al., 2010; Rabin et al., 2018, 2019), the synthesis and release of neurotrophins (Coelho et al., 2013, 2014), and cerebral (Burdette et al., 2010; Bliss et al., 2020) and peripheral blood flow (Scicchitano et al., 2019; O’Brien et al., 2020; Pasek et al., 2020), while also eliciting a positive systemic inflammatory effect (Jensen et al., 2019; De la Rosa et al., 2020). Thus, there is growing support that, at minimum, exercise has the ability to delay the onset of AD and related vascular conditions such as small-vessel-type ischemic stroke and cardiovascular disease (Ngandu et al., 2015; Gallaway et al., 2017; Barnes and Corkery, 2018; Nystoriak and Bhatnagar, 2018; Wardlaw et al., 2019; Alty et al., 2020; Pasek et al., 2020), which African Americans experience at a disproportionately higher prevalence (Soden et al., 2018; Benjamin et al., 2019; El Husseini et al., 2020). While the benefits of regular exercise are known, many adults are not meeting the recommended amount (Benjamin et al., 2019; Centers for Disease Control and Prevention, 2021). For instance, the South has the highest prevalence of physical inactivity compared to other US regions (Centers for Disease Control and Prevention, 2021). Alabama is the 4th least active state (Figure 2F), and African Americans residing in Alabama self-reported physical inactivity at a prevalence of 34.3% (Centers for Disease Control and Prevention, 2021). Perhaps as a direct consequence, Alabama exhibits the 2nd highest AD mortality rate (CDC, 2022e: 50.8). Interventions are typically designed to circumvent these outcomes by including exercise prescriptions consisting of aerobic and/or resistance exercise, while also fostering exercise adherence. It has been shown that aerobic exercise increased serum BDNF in African Americans with MCI, but this improvement was only seen in non-*APOE4* carriers (Allard et al., 2017). This suggested that genotype is an important factor when examining the efficacy of exercise interventions aimed at ADRD risk reduction. This has garnered additional support via a report that aerobic exercise in older African Americans provided no improvement in a hippocampus-related assessment of generalization following rule learning in a high-genetic risk group (Sinha et al., 2020). Interestingly, however, it has been stated that *APOE4* status influences the associations between exercise and ADRD risk such that exercise has a greater ability to protect among *APOE4* carriers (Rovio et al., 2005; Kaufman et al., 2021). Taken together (Rovio et al., 2005; Allard et al., 2017; Sinha et al., 2020), exercise is still beneficial for genetically at-risk individuals, but the most efficacious exercise prescription has yet to be elucidated. Notably, there is currently limited understanding concerning the ability of resistance exercise to improve vascular health with the goal of reducing ADRD risk (Gallaway et al., 2017; Barnes and Corkery, 2018; Landrigan et al., 2020), especially within the African American population (Shin and Doraiswamy, 2016). However, a recent meta-analysis (Coelho-Junior et al., 2020) suggested that resistance training likely improves cognition, but there was no available data regarding the impact of genotype or race/ethnicity. Therefore, this critical gap presents a promising future direction with opportunities for high impact discoveries.

Diet and exercise along with cognitive training and monitoring vascular risk factors may maintain or improve cognitive function in older adults who may have increased risk of developing cognitive decline or dementia. This is supported by results from the Finnish Geriatric Intervention Study to Prevent Cognitive Impairment and Disability (FINGER) study (Ngandu et al., 2015). The World-Wide FINGER network^2^ is working to replicate results with larger and more diverse populations, including the US POINTER Study (NCT03688126).

#### Emotional Wellbeing

The treatment of mood disorders with psychotherapy may serve as a protective factor for vascular health. Several studies have shown that mood disorders predict vascular dysfunction (Frasure-Smith and Lespérance, 2008; Fiedorowicz et al., 2011). Mood disorders such as anxiety and depression may increase the chance of adopting behaviors such as smoking, sedentary lifestyle, and issues with medication adherence (Abed et al., 2014). Depression has been found to impact vascular health (Valkanova and Ebmeier, 2013). Depressed individuals presenting with simultaneous symptoms of anxious distress appear to be at an even higher risk compared to other depressed individuals (Rowan et al., 2005; Almas et al., 2015). Patients with established cardiovascular disease and mental health comorbidities struggle with medication adherence and adopting healthy lifestyle recommendations as well as experience greater disease progression (Ryder and Cohen, 2021). Psychotherapy can potentially serve as a preventative measure for vascular dysfunction through improving behaviors linked to protective factors of vascular dysfunction. Psychotherapy has been effective in improving vascular outcomes for patients with hypertension through adherence to treatment (Ma et al., 2014). Meta-analyses of randomized control trials supported that mental health treatments led to improvements in depression and anxiety, in addition to reductions in coronary heart disease events and cardiovascular mortality (Rutledge et al., 2013; Richards et al., 2018). To prevent the negative cardiovascular consequences of mood disorders, appropriate mental health screening should be conducted by providers (Ryder and Cohen, 2021). The treatment of mood disorders has an overall positive impact on vascular health and functioning.

Non-pharmaceutical interventions for AD have been found to be beneficial preventative measures. Social support can serve as a protective factor for AD. In a study that examined positive and negative effects of social support on the development of AD, positive social support from children was associated with reduced risk of developing dementia (Khondoker et al., 2017). Children may be in a unique position to be impactful members of an elder’s psychological health and should be incorporated into their support system. In a study that examined the relationship between individual forms of social support with early AD vulnerability and cognitive functioning, social support in the form of supportive listening was associated with greater cognitive resilience (Salinas et al., 2021). Social support is one non-pharmaceutical, early intervention that may lead to better outcomes for individuals with ADRD.

#### Hormone Replacement

Midlife aging is a critical time for preventing and delaying neurodegeneration (Mishra et al., 2022). Studies have shown that deprivation of estrogen as well as testosterone relate to cognitive decline in rats, with obesity accelerating the process (Pratchayasakul et al., 2015; Chunchai et al., 2019). The loss of control effect that estrogen has on brain glucose metabolism during the menopausal transition in women, accelerated by *APOE4*, creates a bioenergetic crisis leading to neurodegeneration (Rahman et al., 2019; Mishra et al., 2022). The sex disparities seen in AD patients could be explained by the neuroprotective nature of estrogen in females and the changes in estrogen expression that occur at or around the time of menopause (Snyder et al., 2016; Rahman et al., 2019). Estrogen replacement therapy has potential as a treatment for women with AD but more studies are needed to determine the effects of the stages of the menopausal transition and ultimately if the therapy would be beneficial (Smith et al., 2010; Guo et al., 2020; Song et al., 2020).

#### Oxygen Therapy

As stated above, there are currently limited options available to improve the prognosis of AD, and thus, researchers continue to attempt to understand the efficacy of various off-label treatments such as hyperbaric oxygen therapy (HBOT), notably in trials aimed at promoting neuroplasticity and improving neurocognitive function in humans (Harch and Fogarty, 2018; Xu et al., 2019; Chen et al., 2020; Gottfried et al., 2021; Marcinkowska et al., 2021; Shapira et al., 2021; Somaa, 2021). The effects of this specific treatment have also been investigated via various animal models to reveal HBOT has the ability to reduce neuroinflammation (Shapira et al., 2021), reduce hippocampal neuronal apoptosis (Zhao et al., 2017), and promote neuro- (Zhang et al., 2010) and angiogenesis (Yang et al., 2017). Accordingly, there is rationale to examine HBOT implementation due to the synergistic effects of vascular disease and AD; HBOT might delay or prevent vascular disease and AD via the alleviation of both cerebrovascular occlusion and the resulting cerebral hypoperfusion (Zhang and Le, 2010; Harch and Fogarty, 2018; Xu et al., 2019; You et al., 2019; Gottfried et al., 2021; Shapira et al., 2021; Somaa, 2021). Typical administration of HBOT consists of inhaling 97–100% oxygen under a pressure greater than 1 atmospheric absolute (ATA) (Tibbles and Edelsberg, 1996; Carson et al., 2005), which, in theory, increases the concentration of oxygen dissolved in the plasma as well as arterial saturation to subsequently improve tissue hypoxia (Xu et al., 2019; Fischer and Barak, 2020). In practice, it has been shown that 60 sessions of HBOT (5 d⋅wk^–1^, 90 min of exposure, 100% O_2_ at 2 ATA) induces angiogenesis as demarcated by increases in cerebral perfusion and velocity of blood flow (Hu et al., 2014; Tal et al., 2017). These vascular function/structure benefits have fostered investigations of HBOT pertaining to improved cognitive outcomes (Tal et al., 2017; Zhao et al., 2017; Xu et al., 2019; Gottfried et al., 2021; Marcinkowska et al., 2021; Shapira et al., 2021; Somaa, 2021). For instance, following a similar HBOT exposure prescription (Tal et al., 2017), six elderly patients with significant memory loss exhibited increases in CBF in multiple brain areas as well as improved global cognitive scores (memory, attention, and processing speed were most ameliorated) (Shapira et al., 2021). Additionally, in a large sample of patients diagnosed with vascular dementia, it was revealed that HBOT (5 d⋅wk^–1^, 60 min of exposure, 100% O_2_ at 2 ATA) plus 5 mg⋅d^–1^ of donepezil hydrochloride resulted in higher mini-mental state examination (MMSE) scores, a commonly used cognitive impairment screening test, post treatment than the control (5 mg⋅d^–1^ of donepezil hydrochloride) group (19.8% vs. 9.7%, respectively) (Xu et al., 2019). Further, within this investigation (Xu et al., 2019), it was reported that humanin was increased to a greater extent in HBOT condition compared to the control group (17.4% vs. 13.2%), and of interest, the humanin levels were positively correlated (*r* = 0.409) with the MMSE scores. Overall, the understanding of HBOT to alleviate symptoms of AD via vascular improvements remains in its infancy, but the currently available data is undoubtedly promising, and warrants continued investigation. Future work remains particularly needed to uncover potential race/ethnicity and genetic based differences in responses as well as the efficacy of combining HBOT with other suspected AD preventative care strategies such as diet and exercise.

## Health Disparities for Prevention

### Rural Health Disparities

Some rural Americans, particularly individuals living in the rural South, face greater health disparities compared to both their urban counterparts as well as individuals in other rural areas in the United States (Murray et al., 2006; Miller and Vasan, 2021). The rural mortality penalty is a term used to describe the increased mortality rate observed in rural areas in certain parts of the United States compared to non-rural populations. Southern rural areas, particularly Appalachia, the Mississippi Delta Region, and the Alabama Black Belt have the lowest life expectancies in the country, and this trend has persisted for the last five decades (Singh and Siahpush, 2014; James et al., 2018). While mortality rates are higher in all areas in the South, mortality rates in rural areas in the East South Central Region (e.g., Kentucky, Tennessee, Mississippi, and Alabama) have the highest rates of mortality (Murray et al., 2006; James et al., 2018). Additionally, lower SES and higher rates of poverty correlate to poorer health outcomes. Non-metropolitan areas in Alabama have a poverty rate of 20.6%, which further increases disparities in access to quality healthcare in this population (Rural Health Information Hub, 2022), as shown in Figure 2G. These higher mortality and poverty rates in these underserved Southern rural communities are due to long-standing structural and social barriers to good health, education, jobs, and income/wealth. These communities have greater barriers to accessing quality health care as individuals have to travel longer distance to physicians’ offices, do not have access to public transportation, are more likely to be uninsured, and there are fewer physicians per capita (Miller and Vasan, 2021). The rural South has the fewest number of physicians relative to population, and according to data collected from the Health Resources and Services Administration, in non-metropolitan regions in Alabama, there are only nine physicians per ten thousand people (Miller and Vasan, 2021; Rural Health Information Hub, 2022). The prevalence of health professional shortage in primary care (Figure 2G) and dentistry (Figure 2H). Furthermore, the University of Alabama at Birmingham Alzheimer’s Disease Center is the only academic center for the specialized care of patients with AD in the state. A lack of support and resources for both patients and caregivers in addition to rural Americans having less frequent interactions with healthcare providers all likely contribute to this population receiving a diagnosis of AD at later stages of cognitive decline than their urban counterparts (Rahman et al., 2021). These observations are why it is vital to address the disparities in care and resources present in rural communities, particularly in states like Alabama, where over one million people reside in rural areas of the state (Rural Health Information Hub, 2022).

### Socioeconomic Status and Environment

Although age is a major factor in the development of vascular dysfunction, dementia, and AD, it does not affect the older populations equally, especially those individuals of disadvantaged backgrounds, due to socioeconomic and environmental contributors. SES, the social ranking of an individual or group that is often measured by the combination of education, income, and employment, frequently reveals discrepancies and inequities regarding resource access, ultimately leading to health disparities (Riley, 2012; American Psychological Association, 2021).

Components of SES have been identified as modifiable risk factors for the development of dementia and dementia-related mortality. For example, when comparing individuals with higher SES to those with lower SES, higher SES people should anticipate to live a much longer amount of time without dementia (Cha et al., 2021). In addition, lower SES with low income and financial stress is associated with increased risk of dementia in older persons, and the correlations are similar to those found in older adults with lower education in the United States (Samuel et al., 2020). Also, caregivers who share a home with AD patients with severe neuropsychiatric symptoms (e.g., aggression and anxiety) while living in lower SES areas had more caregiver stress, further showing that different physical and social environmental factors have distinct effects on the likelihood of AD (Alhasan et al., 2021).

Collectively, the aforementioned examples provide evidence that people of lower SES will suffer major consequences of healthcare outcomes and caregiver expenses. Thus, earlier detection and intervention strategies should be developed to steer preventative and risk-reduction measures for vascular dysfunction, dementia, and AD.

### Accessibility to Healthcare and Quality Food

The incidence of AD in Alabama is compounded by poor health predispositions and barriers to receiving necessary medical care. In 2019, the Rural Health Information Hub reported that 47.5% of metropolitan Alabama residents and 31.7% of non-metropolitan Alabama residents had low access to healthy food. Low-access areas were defined as having at least 500 people, or 33% of the population, living more than 1 mile (urban areas) or 10 miles (rural areas) from the nearest supermarket (Rural Health Information Hub, 2022). Additionally, in 2018, there were 7 Primary Care Physicians (PCPs) in metropolitan areas of Alabama per 10,000 people with only 5 PCPs per 10,000 in non-metropolitan areas (Rural Health Information Hub, 2022). The prevalence of healthcare disparities and low accessibility to healthy food in Alabamians is shown in Figure 2. A 2015 report by the Alzheimer’s Association further revealed that 51.0% of adults in Alabama aged 45 and over that are experiencing Subjective Cognitive Decline have not talked to a health care provider (ADPH, 2021).

In the South, including in Alabama, we like our food fried (e.g., chicken, okra, and almost anything else), buttery, salty, and/or sweet. Just writing that sentence can make a Southerner hungry. Detaching culture from food is challenging but if there is more awareness about the impact of diet on cardiovascular health and brain health, perhaps we can reduce the risk of ADRD in Alabama. However, saying one should eat better and exercise more is much easier said than done. It requires creating healthy communities (Crook et al., 2019). Healthy communities are walkable, safe, supportive, intergenerational, loving, and have access to healthy food and other essentials. These safe communities are non-violent, supportive, and invite social interaction and physical activity. These healthy communities are structured on the health equity principle of meeting people where they are and they are not judgmental.

## Beyond Alabama

The aforementioned ADRD risk factors are concentrated in Alabama; however, other regions exhibit many of these risk factors and health disparities as well and should be highlighted. As previously stated, African Americans make up 26.8% of the population in Alabama (Census, 2022). However, the proportion of Black participants and other ethnic minorities in the United States population is growing (Tamir, 2021). Furthermore, the population of minority children and youth grew from 33% (1990) to 38% (2000) to 43% (2008) (Johnson and Lichter, 2010). Therefore, ethnicity- and race-related health disparities must be addressed not only in Alabama but across the United States as well.

Lifestyle-related risk factors for ADRD are also present outside of Alabama. For example, only 8.4% of adults in West Virginia met fruit intake recommendations, compared to the American average of 12.3% (Lee, 2022). Obesity rates also vary between regions of the United States with the highest obesity prevalence in the Midwest (34.1%) and South (34.1%) (CDC, 2021c). Alcohol binge drinking prevalence among adults was highest in Montana, Wyoming, Colorado, North Dakota, South Dakota, Nebraska, Minnesota, Iowa, Missouri, Wisconsin, Illinois, Michigan, Connecticut, Massachusetts, Maine, and Hawaii in 2018 (CDC, 2022b). Cigarette use was highest among adults in West Virginia, Kentucky, Louisiana, Ohio, Mississippi, Alabama, Tennessee, Missouri, Indiana, Oklahoma, Michigan, and North Carolina in 2019 (CDC, 2021b). The LGBTQ student population in 2019 was greater than 18% in New York, Connecticut, Alabama, New Mexico, South Carolina, Florida, Nevada, and Vermont (CDC, 2021a), suggesting that there will be a shift in sexuality among the American adult population in the future. In addition, California, Nevada, Texas, Mississippi, Alabama, Georgia, Florida, South Carolina, North Carolina, Maryland, New Jersey, and Massachusetts had the highest rates of HIV diagnosis among adults and adolescents in 2019 (CDC, 2022d). It will be important to understand the association between these risk factors and ADRD so future ADRD research should include lifestyle-related risk factors, gender and sexuality as variables.

Pathological risk factors for ADRD are present in certain ethnic groups that were not highlighted previously in this review due to lower relevance to Alabama. For example, Filipino Americans are known to have a high risk for cardiovascular disease, hypertension, type 2 diabetes, and metabolic syndrome at lower body mass index levels, likely due to diet and genetics (Abesamis et al., 2016). Despite a higher prevalence rate in non-Hispanic White people, among *APOE4* carriers, Asians had a significantly steeper memory decline when compared to White people (Makkar et al., 2020). Asian-Indians are also known to have the highest coronary artery disease rates (Ardeshna et al., 2018). As previously stated in this review, cardiovascular disease is a known ADRD risk factor leading to impairment of the NVU (Vanherle et al., 2020). Pulmonary diseases, a known risk factor for ADRD, exist at high prevalence in states other than Alabama. Patients with chronic obstructive pulmonary disease (COPD) were found to have a higher risk for dementia (Liao et al., 2015). In 2019, the mortality rate due to COPD ranges from 49.9 to 61.4 deaths per 100,000 people not only in Alabama, but Wyoming, Oklahoma, Arkansas, Mississippi, Tennessee, Kentucky, Indiana, and West Virginia as well (CDC, 2022a).

Cardiovascular and lifestyle risk factors for ADRD are also present in countries other than the United States. For example, Asians including Indonesians (Malays and Chinese ancestry), Singaporean Chinese, Malays and Indians, and Hong Kong Chinese have 3–5% higher body fat than White people at any given body mass index (Deurenberg et al., 2002). A study performed in India showed that South Asians are at high risk for obesity-associated cardiovascular disorders thought to be due to low adipokine production, lower lean body mass, and ethno-specific SES factors (Prasad et al., 2011). In Italy, high cholesterol was found in over 40% of the subject population and abnormal low density lipoprotein values were observed in about 30% (Sofi et al., 2005). In Sub-Saharan Africa, rates of overweight and obesity are rising in children and adolescents due to lifestyle and social determinants of health such as physical inactivity, unhealthy diets, SES, and gender (Choukem et al., 2020). The same study also found many cardiovascular risk factors for ADRD such as metabolic syndrome, hypertension, dyslipidemia, diabetes, and glucose intolerance in the 86,637 children and adolescents in the study (Choukem et al., 2020). A nationwide study in France between 2008 and 2013 showed that 38.9% of early onset dementia cases were alcohol-related and 17.6% of cases had an additional alcohol use disorder (Schwarzinger et al., 2018).

Health disparities due to differences in SES can also be seen globally. A study estimating the global, regional, and national burden of stroke showed that age-standardized low-income groups had several times higher stroke incidence than high-income groups (Feigin et al., 2021). A Danish study also revealed societal inequalities, indicating that those of higher SES appear to be diagnosed with dementia earlier (Petersen et al., 2021). Environmental factors such as air quality, toxic heavy metals, and trace elements have also been shown to be risk factors for ADRD (Killin et al., 2016). Bangladesh (76.9 μg/mL) was found to have the worst air pollution in 2020, followed by Chad (75.9 μg/mL) (IQAIR, 2022) based on particulate matter up to 2.5 μm in diameter. A study focusing on the reasons for Finland’s high dementia mortality rate found that environmental factors such as climate leads to production of neurotoxins that contribute to dementia pathophysiology (Eiser, 2017). The same study also found that trace elements such as mercury is also found in Finnish bodies of water, reducing the quantity and effectiveness of glutathione’s ability to protect against neurotoxins (Eiser, 2017).

## Conclusion

Black, Hispanic and Native Americans have a higher incidence of NVU intrinsic and extrinsic risk factors and consequentially ADRD when compared to White Americans (Alzheimer’s Association, 2022). Black Alabamians, in particular, have a higher prevalence of AD risk factors than other Alabama residents, highlighting tangible health disparities that warrant immediate action. These racial, ethnic, and geographic differences, when compounded with additional disparities in the social determinants of health, further increase the risk for early death for Black Americans in Alabama. In this review, we identified vascular risk factors linked to AD as well as pharmaceutical and non-pharmaceutical treatments and prevention practices to slow or prevent neurovascular dysfunction and cognitive decline as demonstrated in Figure 3. However, individuals living in many regions in Alabama face barriers to implementing these lifestyle changes due to a number of factors such as low SES, the southern rural health penalty, food deserts, and decreased access to quality health care. Consequently, individuals living in Alabama, and similar states, are more prone to adopt unhealthy diets and habits including sedentary lifestyles as well as drug and alcohol use. Ultimately, the combined effect of these inequities lead to a higher risk of NVU dysfunction in Alabama. Community engaged investigators will have to give intentional attention to the mistrust for the health care system within AL’s Black American community. Such engagement by investigators and community members will foster broader community involvement in research that is relevant to underrepresented communities. There is a clear and dire need for future ADRD studies to include more diverse populations with a specific focus on the individuals affected by the disparities outlined in this review.

## Author Contributions

NS and AN conceptualized the initial review outline. NS, E-AB, SC, MT, NH, RR, HB, CF, ML, BH, JK, and AN wrote the first draft of the manuscript. NS, E-AB, SC, MT, HB, FT, EC, JK, and AN reviewed and edited the manuscript. All authors read and approved the final draft of the manuscript.

## Author Disclaimer

The views and opinions expressed in this manuscript represent those of the author and do not necessarily reflect those of the National Institutes of Health, or AlzOut.

## Conflict of Interest

The authors declare that the research was conducted in the absence of any commercial or financial relationships that could be construed as a potential conflict of interest.

## Publisher’s Note

All claims expressed in this article are solely those of the authors and do not necessarily represent those of their affiliated organizations, or those of the publisher, the editors and the reviewers. Any product that may be evaluated in this article, or claim that may be made by its manufacturer, is not guaranteed or endorsed by the publisher.

## Footnotes

1
https://www.theriseregistry.org/

2
www.alz.org/wwfingers
